# Recent progress in TiO_2_–biochar-based photocatalysts for water contaminants treatment: strategies to improve photocatalytic performance

**DOI:** 10.1039/d3ra06910a

**Published:** 2024-01-02

**Authors:** Yunfang Liu, Xiaowei Dai, Jia Li, Shaoheng Cheng, Jian Zhang, Yibo Ma

**Affiliations:** a School of Sciences, Beihua University Jilin 132013 China yiboma@beihua.edu.cn; b Department of Reproductive Medicine Center, The Second Norman Bethune Hospital of Jilin University Changchun 130041 China; c State Key Laboratory of Superhard Materials, College of Physics, Jilin University Changchun 130012 China

## Abstract

Toxic organic pollutants in wastewater have seriously damaged human health and ecosystems. Photocatalytic degradation is a potential and efficient tactic for wastewater treatment. Among the entire carbon family, biochar has been developed for the adsorption of pollutants due to its large specific surface area, porous skeleton structure, and abundant surface functional groups. Hence, combining adsorption and photocatalytic decomposition, TiO_2_–biochar photocatalysts have received considerable attention and have been extensively studied. Owing to biochar's adsorption, more active sites and strong interactions between contaminants and photocatalysts can be achieved. The synergistic effect of biochar and TiO_2_ nanomaterials substantially improves the photocatalytic capacity for pollutant degradation. TiO_2_–biochar composites have numerous attractive properties and advantages, culminating in infinite applications. This review discusses the characteristics and preparation techniques of biochar, presents *in situ* and *ex situ* synthesis approaches of TiO_2_–biochar nanocomposites, explains the benefits of TiO_2_–biochar-based compounds for photocatalytic degradation, and emphasizes the strategies for enhancing the photocatalytic efficiency of TiO_2_–biochar-based photocatalysts. Finally, the main difficulties and future advancements of TiO_2_–biochar-based photocatalysis are highlighted. The review gives an exhaustive overview of recent progress in TiO_2_–biochar-based photocatalysts for organic contaminants removal and is expected to encourage the development of robust TiO_2_–biochar-based photocatalysts for sewage remediation and other environmentally friendly uses.

## Abstract 2

(1) The synthesis technologies, characteristics, and advantages of TiO_2_–biochar-based photocatalysts are introduced. (2) Strategies to enhance the activity of TiO_2_–biochar-based photocatalysts are proposed from three systematic aspects.

## Graphical abstract

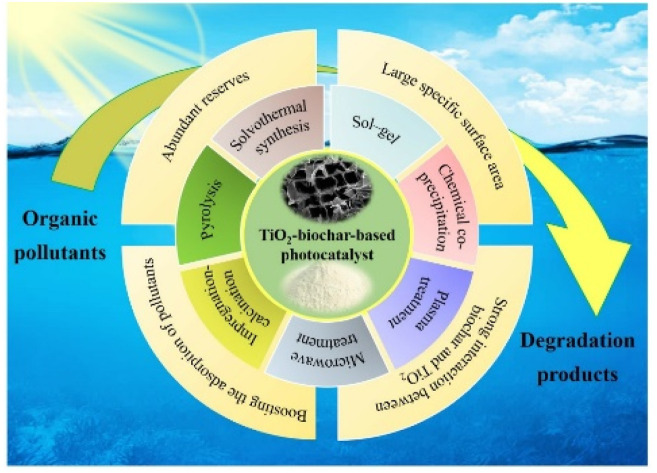

## 1. Introduction

Global concern has been sparked by the fact that water pollution endangers human life and harms ecosystems.^1,2^ It has been reported that numerous toxic organic contaminants, such as phenolic compounds, polymers, cyanides, and detergents, reside in sewage.^3–6^ Several human diseases, even cancer, have been related to organic pollutants in water.^7,8^ Therefore, hazardous contaminants found in wastewater must be appropriately disposed of to protect human health and the environment and to guarantee safe discharge. Most organic pollutants are recalcitrant, toxic, and have weak biodegradability. Thus, they cannot be degraded absolutely *via* traditional treatment techniques (for example, adsorption, biodegradation, and direct burning).^9,10^ Developing a promising treatment technology is paramount for effectively degrading contaminants and ensuring sustainable development. The photoelectrocatalytic water splitting experiment for H_2_ evolution under ultraviolet light in 1972 led to the development of photocatalysis technology.^11,12^ Notably, solar energy is a renewable, clean resource, and photocatalytic technology as an advanced oxidation process offers compelling advantages over conventional strategies for coping with chemical pollutants, such as mild reactions, no additional emissions, high degradation efficiency, and energy savings.^13–15^

The core of photocatalytic degradation of pollutants is to develop highly efficient and inexpensive photocatalysts. Titanium dioxide (TiO_2_) nanoparticles are now regarded as one of the best photocatalytic materials because they have unique electrical and optical properties, are chemically stable, and don't harm living things.^16^ However, the TiO_2_ photocatalyst, which has a broad energy band gap (3.0–3.2 eV) and can only soak up radiation in the near ultraviolet (UV) zone (approximately 5% of the total sunlight spectrum), is limited to visible light applications.^17^ Furthermore, most single-component photocatalysts are provided in the form of tiny particles (such as nanoscale), which effectively increases the specific surface area of the material and aids in enhancing the photocatalytic effect. However, it is more noteworthy that tiny particles can also greatly enhance the intermolecular force, leading to agglomeration effects during the treatment of pollutants, which hinder the effective decomposition of pollutants. At the same time, the recycling and reuse of nanoscale catalysts also face challenges.^18^ To alleviate the drawbacks, integrating photocatalytic materials with substrate materials for modification has been gradually developed and utilized. Representative substrates, including carbon materials (like activated carbon, multiwalled carbon nanotube, and reduced graphene oxide), zeolites, porous polymers, and metal–organic frameworks have been employed to investigate desirable, high-performance photocatalytic materials.^19–21^ Considering the quick recombination rate of photoinduced carriers and the low thermal stability of most substrates, carbon materials are superior in photocatalytic degradation applications.^22^ Carbon–TiO_2_ composites have drawn growing interest as outstanding photocatalysts since carbon materials can serve as electron traps for gathering photoinduced carriers from the photocatalyst surface.^23,24^ At the same time, carbon doping is an efficient approach for introducing impurities into the TiO_2_ lattice, which decreases the band gap and extends visible light absorption.^25^ Biochar is extraordinarily competitive among carbonaceous materials due to its rich in natural resources, non-metallic nature, acid and alkali resistance, and exceptional ecological sustainability, as well as its good physicochemical characteristics, including a high specific surface area, a porous framework, oxygen-containing groups, intrinsic minerals, and active binding sites.^26–28^ These characteristics are advantageous for the binding and growth of photocatalyst nanoparticles, leading to significant loadings, robust stability, and remarkable organic waste photodegradation capabilities.^29,30^ Therefore, biochar is an excellent support for TiO_2_-based catalysts, and in recent years, TiO_2_–biochar-based photocatalysts have been recommended and studied widely.

The combination of TiO_2_ semiconductor and biochar offers several advantages:

(1) Pristine biochar is an appropriate photocatalytic material.^31–34^ Several advanced oxidation processes, like Fenton-like oxidation, photocatalysis, sonocatalysis, and electrocatalysis, have utilized biochar catalysts.^35–38^

(2) As a supporting material for TiO_2_ photocatalysts, biochar has a stable structure and easy recovery.

(3) The porous structure and large specific surface area of biochar provide solid guarantees for the adsorption of pollutants, which can effectively increase the contact between pollutants and TiO_2_ photocatalysts, shorten the carrier migration path, and improve the photocatalytic effect.

(4) Biochar helps create vacancies, acting as photoelectron traps for captured TiO_2_, thereby suppressing the annihilation of photogenerated e^−^–h^+^ pairs. Concurrently, biochar enhances the heat resistance of photocatalysts, reducing their band gap and boosting the response to light sources.^39^

(5) The composite, coupled with biochar and TiO_2_ semiconductor, could present unique and complementary physicochemical properties in favor of water treatment applications.^40^

However, there are also many challenges associated with the combination of biochar and TiO_2_ photocatalysts:

(1) The preparation method of the composite photocatalysts directly affects the bonding strength between biochar and TiO_2_. The nonspecific binding of TiO_2_ photocatalysts may lead to a significant decrease in their effectiveness over time.

(2) The impurities and functional groups in biomass, influenced by factors such as growth environment, can lead to unpredictable performance of each batch of prepared biochar.

(3) The strong biochar adsorption also hinders the recycling of composite photocatalysts.

(4) Improperly applied amounts of biochar shade the light of TiO_2_ photocatalyst, affecting the generation efficiency of carriers.

It is time to review this intriguing topic to recapitulate current developments and, fundamentally, to acquire an in-depth knowledge of the application of TiO_2_ biochar-based materials in photocatalytic decontamination. This review will concentrate on the synthesis methods, physicochemical properties, advantages, and photocatalytic capacity of TiO_2_–biochar-based photocatalysts to remove organic effluent pollutants. Three aspects of some prospective photocatalytic efficiency enhancement strategies are highlighted. Lastly, the significant obstacles and prospects of photocatalysts based on TiO_2_–biochar are presented.

## 2. Biomass for biochar production

Biochar is a unique and renewable carbonaceous substance derived from decomposing biomass residues in oxygen-depleted conditions.^41^ Its porous structure and multitudinous oxygen-containing functional groups on its surface make it suitable for binding pollutant molecules.^42^ In addition, biochar's fitting compatibility and catalytic properties make it an exceptional pollutant removal material for practical application in photocatalysis.^43^

### 2.1 Preparation methods of biochar

It is widely accepted that biomass is the primary source of biochar's feedstock. Carbon-rich biomasses can be regenerated from agricultural and forestry byproducts, garbage, woody products, plants, animal manures, and dietary leftovers.^44,45^ It comprises both lignocellulosic (woody products, agricultural and forestry byproducts, plants) and non-lignocellulosic products (garbage and animal leftovers).^46,47^ Thermochemical techniques can directly convert these numerous forms of biomass into biochar materials.^48^ The most investigated thermochemical treatment methods are oxygen-limited pyrolysis and hydrothermal charring.

Pyrolysis is mainly divided into slow pyrolysis, fast pyrolysis, and flash pyrolysis. Among them, slow pyrolysis has been extensively adopted because of its substantial biochar yield, simple operation, and low cost.^49,50^ Zhang *et al.*^51^ produced biochar by pyrolyzing leftovers from a school cafeteria in oxygen-depleted conditions. The resultant biochar with an even surface was considered an appropriate supporter for attaching BiOBr. Zhang *et al.*^52^ prepared biochar using crop straws as a precursor by pyrolysis at 600 °C for 120 min under limited oxygen conditions. Luo *et al.*^53^ crushed poplar to obtain poplar sawdust and then heated it at 600 °C under N_2_ atmosphere to obtain biochar (BC), which served as a conductive aisle in the ZnFe_2_O_4_/BC/ZnO composite to improve the transportation of photoexcited electrons. Meanwhile, pyrolysis could be used to fabricate biochar doped with other elements. For instance, Wang *et al.*^54^ mixed urea and pine biochar at a particular mass ratio to produce N-modified biochar by pyrolyzing in a vacuum muffle furnace at 500 °C for 120 min. The heating temperature is one of the crucial points for producing biochar *via* pyrolysis. According to previous studies,^55–57^ researchers commonly set the heating temperature at 300–900 °C. Generally, the surface area and pore quantity of biochar increase as the degree of heat rises. However, pyrolyzing biomass at an excessively extreme temperature may cause the carbon skeleton to split, leading to the opposite result.^58,59^ Various pyrolysis procedures will produce biochar with unique physicochemical features and varying application efficacy.^60^ In addition, Shi *et al.*^61^ prepared biochar with different pyrolysis temperatures, which manifested that the photocatalytic ability of biochar rose as pyrolysis temperature decreased. Therefore, an in-depth study of how heating temperature affects biochar's surface features and physicochemical characteristics is worthwhile for the further development of biochar. Overall, modulating heating rates and maintaining an exact response temperature could prove challenging in the pyrolysis process.^27^

The other efficient method for preparing biochar is hydrothermal carbonization (HTC). Typically, HTC is conducted at comparatively lower temperatures and more significant pressures for converting biomass to biochar.^62^ Xu *et al.*^63^ reported that N-doped carbons were generated by HTC at 200–260 °C using sewage sludge, rice husk, and cellulose as precursory biomass materials. Cai *et al.*^64^ employed crushed peanut shells as raw biomass materials by hydrothermal reaction in the existence of iron chloride and hexamethylenediamine (HDA) to produce magnetic HDA-modified biochar. Liu *et al.*^65^ added measured lotus powder to a nitrate solution (La(NO_3_)_3_·6H_2_O and Fe(NO_3_)_3_·6H_2_O were mixed with deionized water) and then carried out a hydrothermal reaction at 180 °C for 90 min. Lastly, C-doped LaFeO_3_/biochar was produced by calcining the sample. Moreover, because carbon content relates directly to ash quantity and pyrolysis temperature, biochar with the desired carbon and ash content can be produced by modulating the hydrothermal temperature.^45^ Compared with pyrolysis, HTC has a higher product yield (up to 88%) and lower energy consumption.^66^ HTC is needed for subsequent processes, such as filtration, centrifugation, and drying, to obtain the desired biochar.

Besides that, the combination of pyrolysis and the HTC process has been conventionally utilized to dope elements into biochar or form biochar-based composites. Talukdar *et al.*^67^ successfully synthesized Ag_3_PO_4_/Fe_3_O_4_ co-doped bamboo-derived activated biochar *via* two-step processes. Specifically, bamboo-derived activated biochar (BAB) was generated by pyrolysis at 500 °C, followed by a wet chemical process to produce an Ag_3_PO_4_/Fe_3_O_4_ co-doped BAB composite. Chen *et al.*^68^ first prepared micro–mesoporous carbon sheets (MMCSs) by pyrolyzing the luffa sponge in a tubular furnace, followed by KOH activation. Then, the obtained MMCSs were mixed with thiourea and dispersed into the distilled water to construct micro–mesoporous carbon sheets doped with nitrogen and sulfur *via* a hydrothermal reaction.

Varying raw materials, production conditions, and modification techniques can alter the surface features of biochar.^49,69–71^ The type of biomass material has the most significant impact on biochar's fixed carbon and ash contents. Biochar with a high ash content is unsuitable for capturing or eliminating contaminants due to its diminished adsorption sites and micropore surface areas.^49,72^ Acid modification can remove the ash and organic matter from biochar, resulting in porous structures and oxygen-containing functional groups.^6,73–75^ Biochar's specific surface area can be increased, and its aromaticity can be altered through alkali modification.^76^ Besides, while metal modification might create additional catalytic active sites, pristine adsorption sites may be occupied, or porous structures may be blocked.^77^ Thus, the design of the doping site and the loading ratio on biochar should be deeply considered. Overall, biochar's surface properties correlate with catalytic performance, so optimizing preparation strategies for biochar catalytic application is imperative.

### 2.2 Adsorption over pristine biochar

The remarkable characteristic of biochar is its adsorption capacity because of its porous nature, enormous specific surface area, and adequate functional groups.^78^ Applications in the adsorption and decomposition of contaminants necessitate a porous material with a high surface area. The specific surface area and porosity may affect the biochar's adsorption capacity and the location of catalytically active sites, thereby determining its performance.^79^ More opportunities for contaminants to connect with biochar surface functional groups and photocatalysts can be obtained in the biochar with a greater surface area and porosity, accelerating adsorption and decomposition rates. Composite photocatalysts are better at breaking down pollutants than single-phase materials because more pollutants stick to the surface of the composite, and more active species can be generated for degradation reactions.^26^ To obtain porous and functionalized biochar, Ma *et al.*^80^ mixed tobacco stem powder with K_2_CO_3_ and low-density polyethylene in a certain mass ratio, followed by pyrolyzing at 900 °C under Ar atmosphere. The produced biochar with an extensive specific surface area and porosity enhanced the potential for adsorption and promoted the separation of photogenerated carriers. In Wu *et al.*'s study,^81^ potassium hydroxide-modified algae-based biochar was successfully fabricated by calcining in the muffle furnace. Briefly, 6 g of prepared biochar was combined with various proportions of KOH. Subsequently, the mixture was calcined in a muffle furnace at 800 °C. The prepared composite material (PEBC) with a weight ratio of 0.5 between biochar and KOH can achieve a maximum adsorption ability of 744.32 mg g^−1^ for sulfamethoxazole, which is far superior to other adsorbents. The test results of PEBCs indicate that the introduction of KOH in the preparation process of macroalgal biochar can effectively modulate the pore structure of the material, increase specific surface area, expand pore volume, and optimize pore size distribution, ultimately obtaining more adsorption sites and promoting pore filling. It is worth noting that the K element in the raw material helps to generate reducing gases (CO and H_2_) during the calcination process, which not only accelerates the formation of the biochar's porous structure but also reduces high-valence metals to low-valence states.^82^

The adsorption mechanism of biochar on pollutants varies at different pyrolysis temperatures, including multilayer reaction, pore filling, mass transfer, π–π stacking interaction, and partition.^83,84^ At low temperatures, electrostatic interactions and hydrogen bonding are the main factors; at high temperatures, the pore filling and π–π stacking interaction dominates.^31^ Specifically, the oxygen-containing functional groups (hydroxyl, carboxyl, carbonyl, and ester groups) on the surface of biochar can rapidly adsorb pollutants and transfer them to photocatalysts through π–π stacking, electrostatic attraction, and hydrophobic interactions.^85^ For example, Yu *et al.*^86^ produced a ZnO/biochar composite photocatalyst that has strong electrostatic adsorption performance and can achieve a removal rate of 95.19% for MB.

## 3. Synthesis strategies for TiO_2_–biochar-based photocatalysts

TiO_2_ is commonly employed as a photocatalyst due to its various advantageous properties, such as effective oxidizing capability, extraordinary chemical stability, and low toxicity.^49,87,88^ Biochar can be used as a supporting material and possesses excellent electrical conductivity, which has great applicability potential in the realm of photocatalytic oxidation.^11^ Combining TiO_2_ with biochar is required to enhance the photocatalytic efficiency of semiconductors to remove organic contaminants. Synthesis strategies for TiO_2_–biochar-based materials have been widely investigated over the past few years. Generally, hydrothermal synthesis is a typical method to prepare TiO_2_ nanoparticles. Regarding biochar, as mentioned above, a popular production process is biomass pyrolysis in oxygen-scarce environments. Here, the preparation strategies of TiO_2_–biochar-based nanocomposites will be introduced by two sectors: *in situ* approaches and *ex situ* synthesis strategies. TiO_2_ growth and loading biochar onto TiO_2_ can be achieved simultaneously using *in situ* synthesis approaches. Typically, *in situ* approaches include solvothermal synthesis, sol–gel, chemical co-precipitation, and pyrolysis. An *ex situ* manufacturing approach attains the biochar combination by reworking the produced TiO_2_ and biochar. *Ex situ* strategies mainly include impregnation–calcination, microwave treatment, plasma treatment, hydrothermal process, and bio-chemical method. TiO_2_–biochar-based photocatalysts synthesized in recent years using these methods are summarized in Table 1.

**Table 1:** Summary of the preparation methods and their photocatalytic degradation activity of recently reported TiO_2_–biochar-based photocatalysts

Photocatalysts	Method	*In*/*Ex situ*	Pollutants	Maximum activity	References
PMBC@TiO_2_	Sol–gel	*In situ*	Sulfadiazine	94.6% 150 min	89
TiO_2_/pBC	Sol–gel	*In situ*	Sulfamethoxazole	91.27% 180 min	75
TiO_2_/biochar	Pyrolysis	*In situ*	Methyl orange	96.88% 150 min	90
Pyrolytic char/TiO_2_	Sol–gel	*In situ*	Phenol	99% 300 min	91
C–TiO_2_	Pyrolysis	*In situ*	Methylene blue	59% 360 min	92
Biochar–TiO_2_	Chemical co-precipitation	*In situ*	Methylene blue	99.2% 240 min	93
TiO_2_–MCDs	Microwave treatment	*Ex situ*	Methylene blue	83% 120 min	94
Biochar–nano TiO_2_	Sol–gel	*In situ*	Toluene	70.1% 300 min	82
Ni-T/AC	Sol–gel	*In situ*	Crystal violet	99% 120 min	95
C–TiO_2_	Microwave treatment	*Ex situ*	Tetracycline hydrochloride	70% 120 min	96
TiO_2_/*Salvinia molesta*	Impregnation–calcination	*Ex situ*	Acid orange 7 dye	90% 240 min	97
TiO_2_/D500	Impregnation–calcination	*Ex situ*	Phenol	77.7% 180 min	11
TiO_2_@BC	Impregnation–calcination	*Ex situ*	Phenanthrene	63.68% 180 min	6
C/TiO_2_	Plasma treatment	*Ex situ*	Formaldehyde	73.7% 180 min	98
N–TiO_2_/N–biochar	Pyrolysis	*In situ*	Methyl orange	97.6% 90 min	99
Biochar–TiO_2_	Impregnation–calcination	*Ex situ*	Methyl orange	97.1% 60 min	100
Zn–TiO_2_/pBC	Sol–gel	*In situ*	Sulfamethoxazole	81.21% 180 min	73
Ag-doped biochar/g-C_3_N_4_/TiO_2_	Solvothermal synthesis	*In situ*	TC-HCl	90.4% 80 min	26
TiO_2_/BC	Sol–gel	*In situ*	Reactive Brilliant Blue KN-R	99.71% 60 min	101
TiO_2_/Fe–C	Solvothermal synthesis	*In situ*	Acetaminophen	100% 360 min	102
Ag/C, N co-doped, Ov-TiO_2_/C	Solvothermal synthesis	*In situ*	2,4-Dichlorophenol	95% 150 min	103
Biochar/TiO_2_	Sol–gel	*In situ*	Sulfamethoxazole	91% 360 min	104
Ag/TiO_2_/biochar	Pyrolysis	*In situ*	Methyl orange	97.48% 60 min	105
N–TiO_2_–Fe_3_O_4_–biochar	Pyrolysis	*In situ*	Methylene blue	99.99% 300 min	106
Biochar–TiO_2_	Impregnation–calcination	*Ex situ*	Diclofenac	90% 120 min	107

### 3.1 *In situ* synthesis

#### 3.1.1 Solvothermal synthesis

**Fig. 1:**
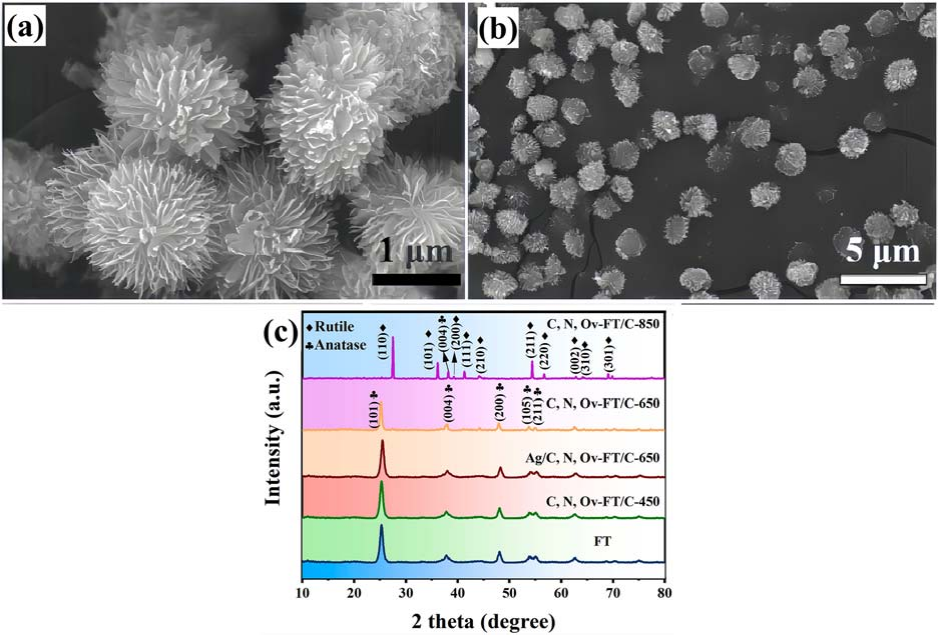
(a) SEM images of flower-like TiO_2_, (b) C, N, Ov-flower-like TiO_2_/C-650. (c) Typical XRD patterns of as-prepared all samples.^103^

The solvothermal method, analogous to hydrothermal, is widely applied to produce catalyst nanoparticles. The solvothermal reaction occurs by dissolving the poorly soluble or insoluble substances to generate crystals under relatively high temperatures and pressure in a closed system.^18^ More importantly, the solvothermal method can yield well-crystalline nanomaterials by moderately tuning preparation conditions (such as temperature and residence time).^108^ Biochar is introduced to TiO_2_ precursor to fabricate TiO_2_–biochar composites *via* the solvothermal method, an effective and typical *in situ* synthesis process. Using solvothermal, Wu *et al.*^103^ successfully generated flower-like C, N co-doped, Ov-TiO_2_/C composites. In detail, the prepared bagasse–urea complex was mixed into tetrabutyl titanate acetic acid solution, and then the mixed solution initiated a solvothermal reaction in an autoclave at 180 °C for 720 min. The final product was sintered at 450 °C in N_2_ atmosphere for 120 min. Fig. 1(a) and (b) is the SEM of the obtained samples. The characterization result showed that the flower-like TiO_2_ particles were successfully decorated on the surface of biochar. In Fig. 1(c), the XRD pattern indicated that as-prepared flower-like TiO_2_ particles show a classical anatase structure and a few weak peaks of the rutile phase. Therefore, the solvothermal method is efficient and convenient for preparing TiO_2_–biochar nanocomposites.

#### 3.1.2 Sol–gel

**Fig. 2:**
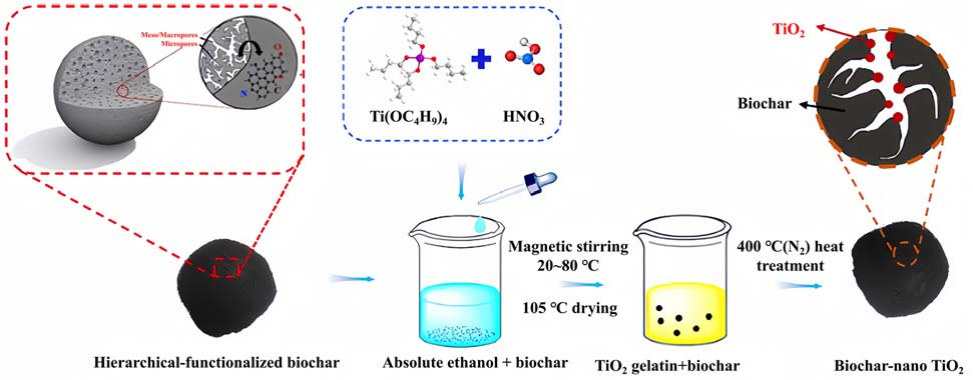
Preparation process of biochar–nano TiO_2_.^82^

Sol–gel refers to the method by which organic metal compounds or inorganic salts are solidified through solution, sol, gel, and heat-treated to become oxides or other solid compounds.^9^ For example, synthesizing of TiO_2_–biochar nanocomposites, the biochar and titanate are stirred evenly in a specific solvent. Then, the pH of the solution is adjusted by nitric acid, and finally, the anti-solvent is added to form a sol. At the same time, the sample can be heated to accelerate the gelation process.^109^Fig. 2 is the flow chart of Guo *et al.*^82^ preparing biochar–nano TiO_2_ cross-linked structure by sol–gel method. He first added rice husk biochar to anhydrous ethanol containing tetrabutyl titanate and diluted nitric acid, then calcined the dried sample at 400 °C under N_2_ protection. The heat treatment process can remove the organic compounds and accelerate the crystallization of sol–gel oxides.^110^ The specific surface area of biochar–nano TiO_2_ is 779.87 m^2^ g^−1^, which is 6.5 times that of pure TiO_2_. This also directly proves that the composite of biochar and TiO_2_ can effectively increase the specific surface area of the sample and further enhance the adsorption performance of the material for pollutants in the photocatalytic process.

#### 3.1.3 Chemical co-precipitation

Chemical co-precipitation is an essential and straightforward strategy for synthesizing metal oxide catalysts, which is performed by combining anions and cations in a solution and producing insoluble solid precipitation.^9^ Fazal *et al.*^93^ synthesized biochar–TiO_2_ composites using a wet precipitation approach. In brief, already prepared biochar (produced from macroalgae by pyrolysis) was dispersed in isopropanol, followed by the dropwise addition of titanium(iv) isopropoxide. The product was then elevated to 80 °C to exhaust the solvent. The sample was then placed in a muffle furnace heated to 400 °C with airflow for 300 min. The SEM images exhibited that the spherical TiO_2_ nanoparticles were evenly modified onto the biochar surface. Also, Herath *et al.*^111^ successfully decorated Fe_2_TiO_5_ nanoparticles on biochar (BC) using a chemical co-precipitation method and a heat treatment process. The XRD examination of Fe_2_TiO_5_ and Fe_2_TiO_5_/BC corroborated the successful deposition of Fe_2_TiO_5_ on the biochar surface.

#### 3.1.4 Pyrolysis

Mian *et al.*^106^ fabricated N-doping TiO_2_–Fe_3_O_4_–biochar composites through one-step pyrolysis of FeCl_3_–Ti(OBu)_4_ laden agar biomass, and the formation of N-doping was achieved *via* under NH_3_ atmosphere. TiO_2_ obtained by hydrolyzing tetrabutyl titanate is *in situ* grown onto biochar, followed by pyrolysis, a feasible synthetic path of TiO_2_–biochar composites. Luo *et al.*^112^ added tetrabutyl titanate into distilled water solution and introduced as-prepared biochar into the system. The solution was vigorously stirred to ensure that biochar was well distributed. Subsequently, the obtained dried TiO_2_/biochar intermediates were activated at 500 °C for 120 min to form TiO_2_/biochar composites.

### 3.2 *Ex situ* synthesis

#### 3.2.1 Impregnation–calcination

Recently, Li *et al.*^6^ effectively utilized the soaking calcination to synthesize TiO_2_@BC *ex situ*, which differs from the *in situ* chemical co-precipitation method, where the already prepared TiO_2_ was directly used. In addition, it can be adopted that TiO_2_ and biochar are mixed by manual mechanical grinding, followed by calcining to prepare the composites. For instance, Lazarotto *et al.*^107^ pyrolyzed the mixture of spent coffee biomass and TiO_2_*via* manual mechanical mixing to prepare biochar–TiO_2_ composites. In the procedure, the biomass was converted into TiO_2_-impregnated biochar. Therefore, employing the impregnation–calcination method to prepare TiO_2_–biochar-based photocatalysts is attractive owing to its easy operation, cost-effectiveness, and reduced refuse production.

#### 3.2.2 Microwave treatment

Microwave treatment is an advanced technology for the *ex situ* synthesis of TiO_2_–biochar-based photocatalysts. Its prominent advantages are short heating treatment time and a safe operation process. The microalgae-based carbon dots (MCDs) were adorned on the outside of TiO_2_ nanoparticles by Vu Nu *et al.*^94^*via* a straightforward microwave procedure. Due to the incorporation of MCDs, the photodegradation efficiency of the TiO_2_–MCDs composite was enhanced. In particular, as photosensitizers of electron acceptors, MCDs can acquire photogenerated electrons and boost the utilization of visible light. Notably, microwave power exerts a significant influence on the properties of synthesized composites.^49^ Oseghe and his coworker^96^ fabricated pine cone-derived C-doped TiO_2_ composites by microwave heating treatment at 600, 800, and 1000 W powers (labeled CT600, CT800, and CT1000, respectively). The studies showed that the BET surface area of composites decreased as microwave power increased. CT800 is better at breaking down tetracycline hydrochloride than other composites because it is more catalytic, has a lower rate of photogenerated carrier annihilation, and has more active species for the redox reaction.

#### 3.2.3 Plasma treatment

**Fig. 3:**
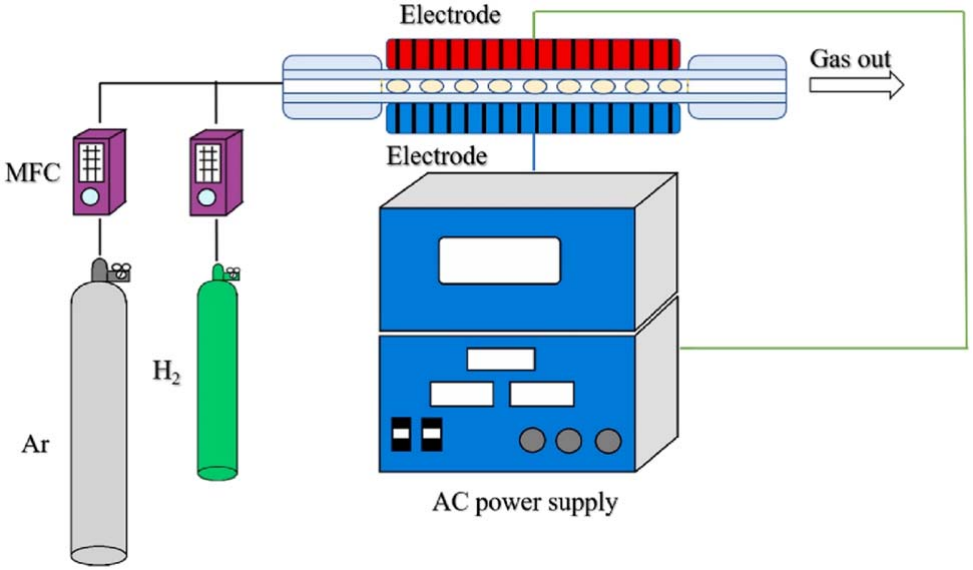
Sketch of the hydrogen dielectric barrier discharge modified system.^98^

Dielectric barrier discharge (DBD), a promising technique for preparing and modifying photocatalysts, has been shown to broaden the utilization of TiO_2_ in the non-UV region.^113^ Several reports have demonstrated that titanium undergoes a transformation of its crystalline and amorphous crystal structures after hydrogen plasma etching, which can promote the utilization of visible light, reduce the binding of photoinduced carriers, and facilitate elemental doping, thus having an extended application in environmental protection.^114^ Recently, Tan *et al.*^98^ successfully synthesized bamboo carbon/TiO_2_ composites by H_2_ plasma using the DBD method (Fig. 3). The results showed that the composite with a 5% bamboo carbon weight ratio has a more meso/microporous structure, narrower band gap width, and lower electron–hole complexation rate and exhibits a good removal effect on formaldehyde.

#### 3.2.4 Other methods

Some new protocols for preparing TiO_2_–biochar-based photocatalysts, such as the hydrothermal process and bio-chemical method, have been developed for *ex situ* synthesis. Recently, Alomairy *et al.*^32^ adopted the bio-chemical method for synthesizing biochar-modified TiO_2_/CoFe_2_O_4_ core–shells. The primary process of integrating biochar with the TiO_2_/CoFe_2_O_4_ core–shell is ultrasonic irradiation, which is considered a feasible and effective tactic. Characteristic results displayed that biochar is conducive to reducing the agglomeration of TiO_2_/CoFe_2_O_4_ core–shell nanoparticles. Sánchez-Silva *et al.*^115^ prepared hydrochar–TiO_2_ composite (HC–TiO_2_) *via* a hydrothermal process using *Byrsonima crassifolia* stones (BCS) as biomass materials. Photodegradation experiments showed that HC–TiO_2_ can achieve 77% removal efficiency of crystal violet (20 mg L^−1^) within 90 min under UV light. Also, the reusability experiments indicated that the HC–TiO_2_ composite has excellent stability for 5 times cycles.

## 4. Advantages of TiO_2_–biochar-based materials in photocatalytic degradation

We summarized some of the latest TiO_2_–biochar-based photocatalysts and TiO_2_–non-biochar-based photocatalysts in Table 2. The results indicated that TiO_2_–biochar-based photocatalysts generally exhibited superior photocatalytic performances and larger specific surface areas than TiO_2_–non-biochar-based photocatalysts. In detail, the advantages of TiO_2_–biochar-based materials in photocatalytic degradation can be embodied in five aspects as follows:

**Table 2:** Comparisons of the photocatalytic properties of TiO_2_–biochar-based photocatalysts and TiO_2_–non-biochar-based photocatalysts

Photocatalysts	Biochar-based/non-biochar based	Light source	Pollutants	Dosage (mg)	Efficiency	*S*_BET_ (m^2^ g^−1^)	*M* a (mg g^−1^)	References
Au/TiO_2_/BCP	Biochar-based	400 W mercury lamp	Tetracycline	8	98.4% (180 min)	1291.1	492	34
			40 mg L^−1^ (100 mL)					
AC/TiO_2_	Biochar-based	UV light	17α-Ethinylestradiol	5	96.23% (120 min)	34.28	11.5	125
			6 mg L^−1^ (10 mL)					
TiO_2_/BC	Biochar-based	30 W UV lamp	Bisphenol A	100	97.6% (60 min)	821	48.8	126
			50 mg L^−1^ (100 mL)					
BC/A/R-TiO_2_	Biochar-based	300 W xenon lamp	Tetracycline	100	99.5% (120 min)	315.8	124.4	127
			50 mg L^−1^ (250 mL)					
TiO_2_/CNTs	Non-biochar-based	1000 W xenon lamp	Methylene blue	200	86.95% (180 min)	—	13	128
			30 mg L^−1^ (100 mL)					
TiO_2_-graphene	Non-biochar-based	250 W mercury lamp	Phenol	300	89.56% (240 min)	62.728	8.9	129
			10 mg L^−1^ (300 mL)					
N-doped TiO_2_/SiO_2_	Non-biochar-based	16 W LED light	Methylene blue	100	72% (720 min)	115.4	—	130
			1 × 10^−5^ M (100 mL)					
TiO_2_/MXene	Non-biochar-based	500 W mercury lamp	Methylene blue	10	96.44% (60 min)	60.47	115.7	131
			60 mg L^−1^ (20 mL)					
ZIF-67/F–TiO_2_	Non-biochar-based	500 W xenon lamp	Tetracycline	30	87% (60 min)	73.93	58	132
			20 mg L^−1^ (100 mL)					

a *M* (mg g^−1^) is denoted as the pollutant mass removed by per unit mass of photocatalyst.

### 4.1 Abundant reserves

Biomass contains nearly all of the living things on the planet and the biological material produced, processed, and expelled by them.^116^ Its broad existence and plentiful stocks ensure the creation of TiO_2_–biochar composites. Therefore, biochar has a conspicuous advantage in feedstock costs compared to other carbon materials and serves an essential function green energy. Moreover, TiO_2_ nanomaterials that are cost-effective and have low toxicity can achieve a promising and long-term application in photocatalysis. Consequently, from the perspective of reserves, TiO_2_–biochar materials are recommended for cheap manufacturing and a green economy.

### 4.2 Large specific surface area

TiO_2_–biochar-based nanocomposites derived from biomass materials as carbon sources possess large specific surface areas because of biomass carbon's natural hierarchical porous microstructure.^117^ Specifically, macropores promote rapid fluid movement, whereas mesopores and micropores ensure complete interaction between contaminants and the photocatalyst surface. Also, biochar with a more porous structure makes TiO_2_–biochar-based photocatalysts better at capturing light by taking advantage of how light reflects and scatters in the pores. More importantly, the surface active centers and exposed atoms of the materials increase with specific surface areas. These active sites and unsaturated atoms help stabilize the catalytic process's intermediates and reduce the activation barrier.^118^ In our previous study,^34^ we reported a novel Au nanoparticles/TiO_2_ nanorods/biochar (Au/TiO_2_/BCP) photocatalyst with a massive specific surface area of 1291.10 m^2^ g^−1^. Photodegradation results demonstrated that Au/TiO_2_/BCP reached a removal efficiency of 98.4% for degradation tetracycline, and per unit mass can remove 492 mg tetracycline. Therefore, the pollutant degradation rate is considerably improved using TiO_2_–biochar-based photocatalysts.

### 4.3 Boosting the adsorption of pollutants

Since the photocatalytic oxidation process happens on the exterior of the photocatalyst, a superior surface-based adsorption performance is necessary for the efficient destruction of contaminants.^119^ Biochar has more surface functional groups (such as hydroxyl and carboxyl groups) than activated carbon, which is one of the most pivotal factors influencing adsorption efficacy.^120^ As a result, more pollutants in wastewater can be adsorbed and decomposed near the surface of photocatalysts, thereby achieving high photocatalytic efficiency, which is a crucial step in developing feasible applications of photocatalysts in wastewater treatment.

### 4.4 Strong interaction between biochar and TiO_2_

Owing to the strong interaction between carbon materials and metal Ti atoms, biochar and TiO_2_ can form a close contact structure. This improves the material's physical and chemical properties and the separation and migration of photoinduced charge, enhancing the lifetime of carriers and making them less likely to recombine.^121^ For instance, Zhai *et al.*^122^ manifested that TiO_2_/bio-char had higher abilities of adsorption and decomposition for methylene blue than the bio-char, for which this composite existed a benign interaction between TiO_2_ and bio-char and offered an enhanced electron transport efficiency.

### 4.5 Accelerating charge migration in the TiO_2_–biochar-based composites

**Fig. 4:**
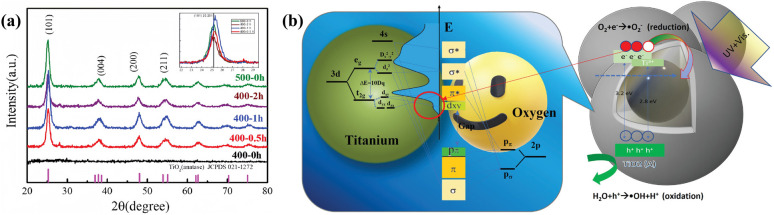
(a) X-ray diffraction patterns of nanospheres. (b) The mechanism of mobile electron transfer and hole increase under UV-vis illumination.^124^

In a TiO_2_–biochar composite photocatalysis system, biochar with appropriate conductivity can serve as an electron trap and a transit channel, promoting the separation and migration of photoinduced charge carrier pairs of TiO_2_ and boosting the redox reaction. Furthermore, more reactive oxygen species can be generated by TiO_2_–biochar composites, thereby enhancing the photocatalytic activity.^123^ Hsu *et al.*^124^ successfully deposited TiO_2_ on the carbon nanospheres to construct carbon@titania yolk–shell nanostructures. The XRD results (Fig. 4a) indicated that TiO_2_ hollow nanospheres prepared by sol–gel and calcination possess anatase structure. As shown in Fig. 4b, owing to the presence of carbon nanospheres, photoexcited electrons on the TiO_2_ can be transferred, effectively inhibiting the recombination of electron–hole pairs.

## 5. Strategies for improving photocatalytic performance of TiO_2_–biochar-based photocatalysts

### 5.1 In terms of biochar

The improved strategies for biochar can be concluded in two aspects. For one thing, the preparation temperature of biochar was found to have significant effects on its features and the performance of the TiO_2_–biochar photocatalyst. Xie *et al.*^133^ fabricated biochar utilizing three distinct pyrolysis temperatures (300–700 °C). The results demonstrated that the TiO_2_–biochar prepared at 500 °C displayed the most effective purification of manufactured rainwater, as the stable tubular structure of the biochar facilitated TiO_2_ nanoparticle attachment. For another, the surface modification of biochar is a vital point in achieving a uniform dispersion of nano-TiO_2_ photocatalysts. Acid oxidization can erode the biochar surface, introduce functional groups, and expose more adsorption sites conducive to controllable superficial growth.^104,134^ The TiO_2_ particles loaded on the surface of acid-treated biochar are smaller than those on untreated surfaces. This is because acid oxidation can effectively reduce the clumping of nanoparticles.^82^ Besides, biochar with a hierarchical porous structure can be achieved *via* activating an alkali, such as KOH.^101,135^ As a result, selecting suitable preparation conditions and functionalized treatment technology for biochar is an attractive tactic for enhancing its physical and chemical characteristics.

### 5.2 In terms of TiO_2_

**Fig. 5:**
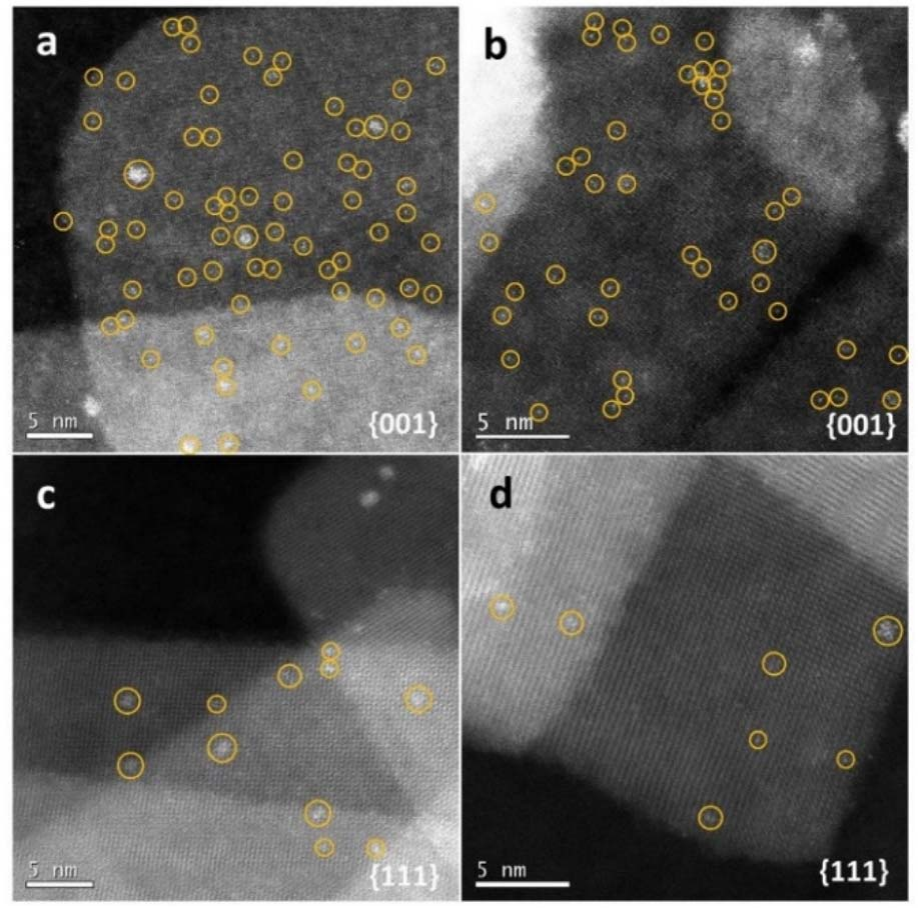
Photo-deposition of Pt on different facets after one minute of illumination. (a and b) HAADF images of Pt deposited on {001} facets. (c and d) HAADF images of Pt deposited on {111} facets.^138^

The exposed facets of nanocrystals can easily affect chemical reactions on the external layers of photocatalysts during the photodegradation process. More importantly, different crystal facets present different surface atomic arrangements and crystallographic orientations, which determine the spatial charge separation between facets and reactive sites and diverse molecule absorption abilities.^136,137^ Qu *et al.*^138^ investigated the photocatalytic water splitting performance of anatase TiO_2_ with predominant {001} facets and {111} facets, showing that the {001} facets displayed higher activity. The substantial amount of surface undercoordinated Ti atoms and the surface atoms' strained configuration are advantageous for water adsorption and dissociation. Besides, as shown in Fig. 5, *in situ* photochemical probing reactions have corroborated that more nucleation sites exist on the {001} facets of anatase TiO_2_. In addition, utilizing the strong oxidation ability of TiO_2_ (001) facets, Li *et al.*^139^ successfully designed a MoS_2_/Au/TiO_2_ heterogeneous photocatalyst with highly-exposed (001) facets of TiO_2_. Notably, the photogenerated holes in this composite are primarily on the large TiO_2_ (001) facets. This means that more oxidation sites are exposed, which speeds up the photocatalytic removal process. Thus, to boost the photocatalytic capacity of TiO_2_–biochar-based photocatalysts, it is crucial to tune the exposed facets of TiO_2_ nanocrystals during the manufacture of TiO_2_–biochar-based composites.

**Fig. 6:**
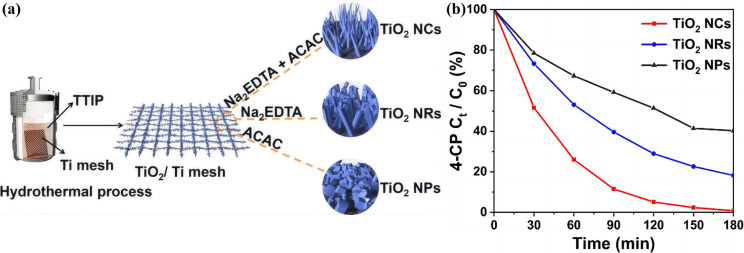
(a) Synthesis scheme of TiO_2_ catalysts with different structures by a Na_2_EDTA-assisted hydrothermal approach. (b) The percentage of 4-CP degradation.^141^

**Fig. 7:**
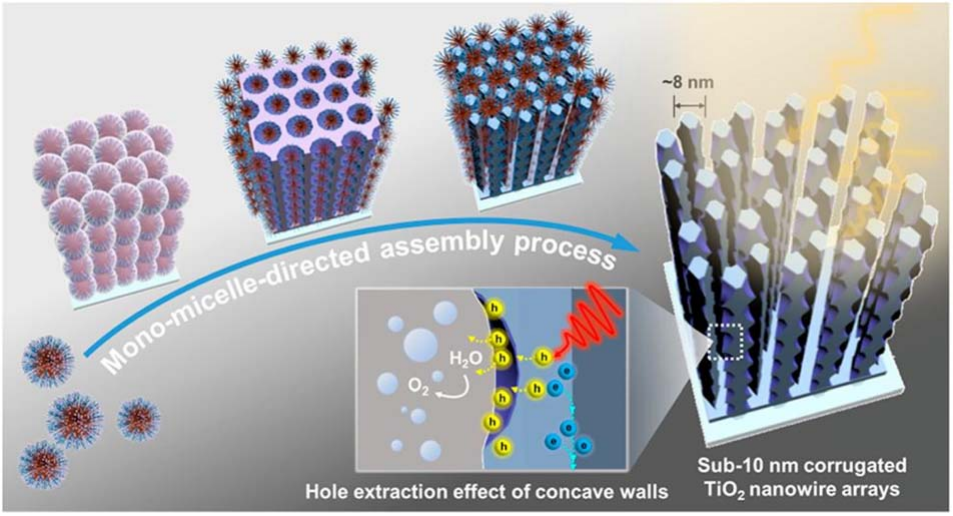
Schematic illustration of the formation process for the ultrathin corrugated TiO_2_ nanowire arrays synthesized *via* the monomicelle-directed assembly approach.^142^

On the other hand, controlling the configuration of TiO_2_ nanocrystals at the microscopic level cannot merely accelerate the movement of charges but also facilitate mass delivery, resulting in an enhanced photocatalytic performance for the disintegration of organic toxins.^140^ Herein, Song *et al.*^141^ synthesized TiO_2_ nanomaterials with three different morphologies (nanocones, nanorods, and nanoparticles) using a Na_2_EDTA-assisted hydrothermal technique (Fig. 6a), which were used as catalysts for the photoelectrocatalytic (PEC) removal of pollutants. The outcomes of the degradation experiment revealed that the nanocone-shaped TiO_2_ (99.3%) had superior PEC performance compared to the nanorod-shaped TiO_2_ (82.8%) and nanoparticle-shaped TiO_2_ (62.7%) (Fig. 6b). This makes it easier to complete the mass transfer between catalysts with a conical array structure. Notably, the nanocone structure possesses a potent built-in electric field that facilitates carrier separation and migration, a prerequisite for producing active oxides. In addition, Zhang *et al.*^142^ synthesized well-aligned sub-10 nm TiO_2_ nanowire arrays with adjustable corrugated structures by a distinctive mono micelle-directed assembly tactic (Fig. 7). Characterization results demonstrated that ultrathin corrugated TiO_2_ nanowire arrays could separate photocarriers, transfer charges, and activate surface holes all at the same time. The remarkable optoelectronic behaviors are predominately determined by the exceedingly large surface area and rapid charge separation capability of the as-prepared, unusual TiO_2_ arrangements. Consequently, it is desirable to finely manipulate the configuration of TiO_2_ nanocrystals to ameliorate the photocatalytic efficiency of TiO_2_–biochar-based photocatalysts.

### 5.3 The synergistic effect of biochar and TiO_2_

Finding the optimal amount of carbonaceous material to add to TiO_2_ when making TiO_2_–biochar-based photocatalysts is important for biochar and TiO_2_ to work together and for the photocatalytic activity to improve. Xie *et al.*^133^ fabricated TiO_2_–biochar composites using three various TiO_2_–biochar mass proportion values by combining the necessary quantities of TiO_2_ and biochar. The research results indicated that the purification effect of the TiO_2_/biochar composite on artificial wastewater decreases with a decrease in mass ratio. This is because low mass ratio biochar blocks TiO_2_, weakens the transmission between light and TiO_2_, and loses photocatalytic efficiency.^143^ However, too high a quality ratio can backfire, and the advantages of biochar materials cannot be fully utilized. Lu *et al.*^90^ made several kinds of TiO_2_/biochar composite catalysts with varying biochar-to-Ti mass ratios. They designated it as CT*x*, where *x* (*x* = 0.1/1, 0.2/1, 0.5/1, 0.8/1, 1/1) indicated the biochar-to-Ti mass ratios. In the photocatalytic oxidation experiments of methyl orange, CT0.2/1 showed the best effect, with a decolorization rate of nearly 97% and a mineralization rate of nearly 83%. It indicates a synergistic effect between biochar and catalyst, and adding a proper amount of biochar can promote the photocatalytic process.

Besides that, TiO_2_–biochar nanocomposites with controllable porous microstructure and uniform morphology can be constructed *via* coupling with the template method, which contributes to the exposure of adequate activity sites for photocatalytic reactions. Wang *et al.*^144^ fabricated hierarchical porous titanium dioxide/carbon nanocomposites (TiO_2_/C) by introducing ice and nanoscaled silica as soft and hard templates for porosity modulating. The as-prepared TiO_2_/C nanocomposites exhibited more effective photocatalytic activity compared to their counterparts lacking templating, which can be assigned to the instructed porous structure and distinctive shape, which offer transfer avenues for electrolytes and photogenerated charge carriers, thereby enhancing the effectiveness of the degradation process.

## 6. Conclusions and future perspectives

In summary, TiO_2_, as an excellent photocatalyst, has been applied for almost half a century. To overcome the drawbacks of TiO_2_ and further broaden its application range, biochar, a novel carbonaceous material with fascinating physicochemical properties and environmental benignancy, is employed to couple with TiO_2_ in the field of photocatalysis. Mainly, TiO_2_–biochar-based composites have been extensively used to photodegrade organic contaminants in wastewater. Outstanding photocatalytic performance is required for TiO_2_–biochar-based composites to be extensively applied and developed quickly. The reasoned design and manufacturing of TiO_2_–biochar-based photocatalysts provide a potent means of enhancing the efficiency of light capture and photogenerated carrier separation. Through substantial research, multiple techniques and strategies have been adopted to prepare and modify TiO_2_–biochar-based photocatalysts. In this review, we introduced the characteristics of biochar and summarized the synthesis approaches (including *in situ* and *ex situ* means), advantages, and strategies for optimizing the performance of TiO_2_–biochar-based photocatalysts. However, TiO_2_–biochar-based photocatalysts still need to be adequately explored. Although the distinctive advantages of TiO_2_–biochar composites should be approved, the properties of TiO_2_–biochar composites should be adequately researched, and the shortcomings of TiO_2_–biochar-based photocatalysts in the application of photodegradation pollutants should be rationally realized. These preliminary investigations are essential for the advancement of photocatalysts based on TiO_2_–biochar. In the following aspects, several significant improvements in the research on TiO_2_–biochar-based photocatalysts can be accomplished.

First, as a dominating feature of biochar materials, the porous structure closely correlates with biochar's adsorption ability and photocatalytic performance. Meanwhile, the definite effect mechanisms have yet to be entirely explored. Thus, follow-up research concerning the mechanism of the porous structure of biochar remains to be done.

Second, regarding the combination of biochar and TiO_2_ nanoparticles, the different loading proportions of TiO_2_ on the surface and porous channels of biochar affecting photocatalytic activity have been extensively studied. Nonetheless, the specific interaction of TiO_2_ load proportions and the as-synthesized composites of physical and chemical properties needs to be more explicit. In the future, the quantitative structure–activity relationship (QSAR) of TiO_2_ load proportions and structural/optical features (*e.g.*, BET, pore volume, pore type, and light absorption ability) of TiO_2_–biochar-based composites is desirable to investigate thoroughly, which has a significant effect on the understanding and reasonable design of high-performance TiO_2_–biochar-based photocatalysts.

Third, the computational calculation for the pathways of TiO_2_–biochar-based photocatalysts degradation pollutants is an effective means to investigate the decomposition process of pollutants. Unfortunately, limited by the fact that it is challenging to build the theoretical calculation model due to the complex structure of biochar, the theoretical calculation of photocatalytic degradation pollutants by TiO_2_–biochar-based photocatalysts has yet to be developed comprehensively. More efforts on the theoretical calculation of TiO_2_–biochar-based photocatalysts are necessary, including the adsorption mechanism between photocatalysts and pollutants, electrons transfer routes, and the minute decomposition pathway of contaminants.

Fourth, although we summarized many different methods for preparing TiO_2_–biochar-based photocatalysts in this review, large-scale manufacture still needs to be improved in real-world applications. Also, the high production costs always limit the industrialization process of TiO_2_–biochar-based photocatalysts. In order to decrease the costs of photocatalytic elimination of organic pollutants in water further using TiO_2_–biochar-based photocatalysts, some appropriate strategies should be extensively investigated.

Fifth, most studies of TiO_2_–biochar-based photocatalysts for the degradation of pollutants were implemented under UV light, although a favorable removal efficiency can be reached. Engineering strategies of TiO_2_–biochar-based photocatalysts should be explored to tune intrinsic physicochemical properties and to expand the response of visible light. Besides, developing multi-functional and highly active TiO_2_–biochar-based photocatalysts is needed to degrade the wastewater that consists of various persistent pollutants. Therefore, further investigation of TiO_2_–biochar-based photocatalysts can focus on adjusting properties and regeneration to adapt to practical applications.

It is worth exploring these aspects we discussed, for which TiO_2_–biochar-based materials have a promising future for photocatalytic degradation. Reasonable design and superior performance for TiO_2_–biochar-based photocatalysts can conspicuously boost the development of wastewater treatment and energy conversion. This review will present a comprehensive cognizance of TiO_2_–biochar-based photocatalysts. In the future, broader application fields of TiO_2_–biochar-based materials could be realized through more research, such as applying them in solar cells, adsorption, and supercapacitors.

## Author contributions

YL: investigation, review, editing, writing—original draft; XD: review and editing; JL: review and editing; SC: investigation; JZ: review and editing; YM: investigation, writing, review, editing, validation, and supervision; all authors read and approved the final manuscript.

## Conflicts of interest

There are no conflicts to declare.

## Supplementary Material
